# Processed data for CHMS 2007–2009: Bisphenol A, phthalates and lead and learning and behavioral problems in Canadian children 6–19 years of age

**DOI:** 10.1016/j.dib.2016.06.017

**Published:** 2016-06-22

**Authors:** Tye E. Arbuckle, Karelyn Davis, Khrista Boylan, Mandy Fisher, Jingshan Fu

**Affiliations:** a Population Studies Division, Healthy Environments and Consumer Safety Branch, Health Canada, Ottawa, ON, Canada; b Department of Psychiatry and Behavioural Neurosciences, McMaster University, Hamilton, ON, Canada

**Keywords:** Bisphenol A, Lead, Phthalates, Behavior, Children

## Abstract

This article presents processed data from an analysis of cross-sectional data from Cycle 1 of the Canadian Health Measures Survey (CHMS) to examine the potential association between urinary concentrations of BPA and phthalate metabolites and child learning and behavioral problems, considering important covariates such as gender, blood lead and environmental tobacco smoke (ETS). These processed data are related to the research on a subset of the children (Arbuckle et al., 2016) [1]. The Strengths and Difficulties Questionnaire (SDQ) outcomes of interest were emotional symptoms, hyperactivity/inattention, and a total difficulties SDQ score, with borderline and abnormal scores grouped together and compared with children with normal scores. Other outcomes studied included reported learning disability, ADD/ADHD (attention deficit disorder/attention deficit hyperactivity disorder) and use of psychotropic medications to treat behavioral disorders in the past month. Data are presented for all children 6–19 years of age combined.

Weighted simple logistic regression estimates for important covariates of each of the outcomes from CHMS Cycle 1 children are reported. Odds ratios based on weighted multiple logistic regression estimates for urinary BPA and phthalate metabolites (including specific gravity as a covariate) and blood lead are presented for the reported outcomes ADD/ADHD, learning disability and psychotropic medications, as well as the SDQ outcomes emotional symptoms, hyperactivity/inattention and total difficulties.

**Specifications Table**

**Table:** 

Subject area	Psychology
More specific subject area	Environmental health
Type of data	Tables
How data was acquired	Survey
Data format	Analyzed
Experimental factors	Restricted to children with environmental chemical biomonitoring data
Experimental features	Applied survey weights
Data source location	Canada
Data accessibility	Data are within this article

**Value of the data**

• Cross-sectional analysis of associations between child levels of BPA, phthalates and lead with indicators of adverse child behavior, controlling for wide set of covariates.
• Generates exploratory data useful for examination in prospective cohort studies.
• Could be compared with similar data from US NHANES for further insight.

## 1 Data

Tables of results of simple weighted regression analysis of important covariates and weighted multiple logistic regression analysis for chemicals examined. Child behavioral outcomes considered were reported learning disability, ADD/ADHD and taking psychotropic medications, as well as Strengths and Difficulties Questionnaire scores for hyperactivity/inattention, emotional symptoms and total difficulties.

## 2 Experimental design, materials and methods

The Canadian Health Measures Survey (CHMS) was designed to collect key information on the health of Canadians using direct physical measurements, collection of blood and urine and household and clinic interviews [2], [3]. The target population for Cycle 1 were individuals between 6 and 79 years of age living in privately occupied dwellings, representing 97% of Canadians. This dataset was restricted to children and adolescents 6–19 years of age (*n*=2097). Information on child behavior, demographic, socioeconomic and lifestyle factors was collected by a questionnaire administered to the parent or guardian of children 6–11 years of age or directly to the child 12 years and older.

Blood was analysed for lead, while urine was analysed for BPA and phthalate metabolites. Creatinine was measured to adjust for urine dilution differences between spot urine samples. Chemical lab measures below the limit of detection were imputed as half the limit of detection.

For children 6–19 years of age, outcomes examined were reports of self-, or in the case of children 6–11 years, parent-reported learning disability (any, ADD or ADHD) and whether any medications used to treat behavioral disorders were taken in the past month. Respondents reported medications by Drug Identification Numbers (http://www.hc-sc.gc.ca/dhp-mps/prodpharma/activit/fs-fi/dinfs_fd-eng.php) which were coded to Anatomical Therapeutic Chemical (ATC) codes (http://www.whocc.no/atc_ddd_index/). One of the co-authors (KB), a clinical child psychiatrist, provided a table of medications potentially used for treating behavioral disorders in children (see Supplemental Material, Table S1 [1]).

For children 6–17 years of age, borderline and abnormal scores from the Strengths and Difficulties Questionnaire (SDQ) (www.sdqinfo.com) were grouped together and compared with children with normal scores for the outcomes emotional symptoms, hyperactivity/inattention, and the total difficulties scores.

Initially, for each outcome of interest, weighted univariate models were considered for each contaminant. Subsequently, potential risk factors were evaluated. Covariates identified through reviews of the literature included child׳s age, body mass index, number of hours slept per night, gender, highest level of household education (secondary school or less vs. at least some postsecondary studies), income adequacy (low/lower middle vs. upper middle/higher income), whether the child fasted prior to specimen collection, and ETS exposure in the home. For children 6–11 years of age only, additional covariates were available and considered: prenatal smoking, birth any time prior to due date, admission to a special neonatal unit or an intensive care unit prior to leaving hospital, and breast feeding (less than 3 months vs. three months or longer), as well as number of days in a neonatal unit, birth weight, and mother׳s age at birth.

Since the CHMS employed a complex, multistage survey design, survey weights were used in statistical modeling to account for the unequal probabilities of selection. Due to the complex sampling scheme of the CHMS Cycle 1 survey, direct calculation of standard errors and confidence intervals were not possible. To that end, Statistics Canada [3] provided bootstrap weights in order to calculate standard errors, confidence intervals and coefficients of variation for each estimate using the bootstrap method.

Weighted simple logistic regression modelling was done for each of the identified covariates and the outcomes (Table A1, Table A2, Table A3, Table A4, Table A5, Table A6, Table A7). Weighted multiple logistic regression models were then run considering the environmental contaminants and other important covariates identified in the simple regressions (Table B1, Table B2, Table B3, Table B4, Table B5, Table B6). For urinary chemicals, creatinine concentration was included in all the multiple regression models as a separate independent variable [4].

In order to determine which of the available variables resulted in the best fit, a stepwise procedure was implemented. The natural-log of the contaminant concentrations was used since the contaminants were lognormally distributed based on the Anderson–Darling test. However since the complex survey design limited the number of degrees of freedom to 11, a stepwise selection method was used to determine which covariates were most significant to improve the model fit. The contaminant and creatinine concentrations were retained in the model, and then other covariates were sequentially added to the model based on the smallest *p*-value (i.e. the most significant variables). This approach facilitated the evaluation of demographic variables one-at-a-time with respect to their p-value, conditional on other variables already in the model. This approach also served to examine the effect of multicollinearity, which could inflate standard errors and provide misleading results. For some models, after examining the main effects, sufficient degrees of freedom were available to evaluate an interaction term between highly significant covariates. Furthermore, to compare models, the model with significant terms and with the lowest value of the Akaike Information Criterion (AIC) was selected. Goodness of fit was assessed using the Hosmer–Lemeshow test. Odds ratios were calculated from weighted multiple logistic regression models for a 1-unit increase in the log of the contaminant concentration (Table C1, Table C2).

The software package SAS (Statistical Analysis System) Enterprise Guide 4.2 was used for statistical analysis. For regression modeling, the software programs BOOTVAR and SUDAAN were used along with the bootstrap weights, in order to correctly calculate such estimates. Finally, for all statistical analysis performed, an inference was deemed significant at *α*=5% unless otherwise indicated.

## Figures and Tables

**Table A1:** Weighted simple logistic regression estimates for covariates from CHMS Cycle 1 with outcome reported ADD (6–19 years of age).

Outcome: ADD							
**Factor (reference category)**	***n***	**Estimate**	**Standard error**	***P*-value**	**Odds ratio (OR)**	**95% CI for Odds ratio**	
Child׳s age	2087	0.14	0.06	0.05	1.15	1.00	1.33
BMI – measured	2081	0.00	0.04	0.97	1.00	0.91	1.09
Gender (males)	2087	1.04	0.58	0.10	2.83	0.79	10.06
Education (secondary school or less)	2031	0.73	0.52	0.18	2.07	0.67	6.45
Income (low/lower middle)	1927	1.04	0.48	0.05	2.83	0.99	8.09
Prenatal smoking (yes)	1038	0.27	0.71	0.71	1.31	0.24	7.10
Born any time before due date (yes)	1047	0.95	0.61	0.18	2.6	0.54	12.39
Special neonatal unit care (yes)	1047	1.24	0.4	0.01	3.47	1.40	8.57
No. of days in neonatal unit	1066	0.01	0.11	0.9	1.01	0.8	1.29
Hours of sleep per night	2087	0.17	0.16	0.31	0.84	0.59	1.21
Birth weight	1039	−0.00	0.00	0.21	1.00	1.00	1.00
Breast feeding (3 months or longer)	1037	−0.77	0.67	0.28	0.46	0.10	2.03
Mother׳s age at birth	1065	−0.03	0.06	0.6	0.97	0.84	1.11
Anyone smoking at home (yes)	2082	1.48	0.49	0.01	4.38	1.49	12.88
Fasted (yes)	2087	−0.14	0.47	0.77	0.87	0.30	2.46

**Table A2:** Weighted simple logistic regression estimates for covariates from CHMS Cycle 1 with outcome reported ADHD (6–19 years of age).

Outcome: ADHD							
**Factor (reference category)**	***n***	**Estimate**	**Standard error**	***P*-value**	**Odds ratio (OR)**	**95% CI for odds ratio**	
Child׳s age	2087	0.000	0.05	0.95	1.00	0.90	1.1
BMI – measured	2081	−0.05	0.04	0.16	0.95	0.87	1.03
Gender (males)	2087	1.31	0.48	0.02	3.69	1.27	10.71
Education (secondary school or less)	2031	−0.11	0.66	0.87	0.90	0.20	3.96
Income (low/lower middle)	1927	0.96	0.45	0.06	2.62	0.96	7.13
Prenatal smoking	1038	1.22	0.35	0.006	3.39	1.55	7.40
Born any time before due date	1047	0.62	0.39	0.140	1.87	0.79	4.39
Special neonatal unit care	1039	NR	NR	NR	NR	NR	NR
No. of days in neonatal unit	1066	NR	NR	NR	NR	NR	NR
Hours of sleep per night	2087	−0.03	0.13	0.83	0.97	0.73	1.29
Birth weight	1039	0.00	0.00	0.78	1.00	1.00	1.00
Breast feeding (3 months or longer)	1037	−0.48	0.79	0.55	0.62	0.11	3.48
Mother׳s age at birth	1065	−0.11	0.07	0.14	0.90	0.77	1.04
Anyone smoking at home (Yes)	2082	0.67	0.5	0.21	1.95	0.65	5.83
Fasted (yes)	2087	0.38	0.33	0.26	1.47	0.72	3.00

NR – Not reported due to unacceptable quality.

**Table A3:** Weighted simple logistic regression estimates for covariates from CHMS Cycle 1 with outcome reported learning disability (6–19 years of age).

Outcome: Learning disability							
**Factor (reference category)**	***n***	**Estimate**	**Standard error**	***P*-value**	**Odds ratio (OR)**	**95% CI for odds ratio**	
Child׳s age	2094	0.04	0.03	0.33	1.04	0.96	1.12
BMI – measured	2088	0.01	0.02	0.6	1.01	0.97	1.05
Gender (males)	2094	0.85	0.31	0.02	2.34	1.19	4.60
Education (secondary school or less)	2038	0.26	0.26	0.33	1.30	0.74	2.3
Income (low/lower middle)	1934	0.92	0.24	0.003	2.50	1.47	4.27
Prenatal smoking (yes)	1043	0.71	0.35	0.07	2.04	0.94	4.42
Born any time before due date (yes)	1052	0.28	0.26	0.30	1.32	0.75	2.35
Special neonatal unit care (yes)	1072	0.82	0.35	0.04	2.27	1.06	4.86
No. of days in neonatal unit	1071	0.01	0.02	0.66	1.01	0.97	1.05
Hours of sleep per night	2094	−0.1	0.1	0.35	0.91	0.73	1.13
Birth weight	1044	0	0	0.86	1.00	1.00	1.00
Breast feeding (3 months or longer)	1042	−0.25	0.33	0.46	0.78	0.38	1.6
Mother׳s age at birth	1070	−0.06	0.04	0.13	0.94	0.86	1.02
Anyone smoking at home (yes)	2089	0.84	0.21	0.002	2.32	1.48	3.64
Fasted (yes)	2094	0.1	0.20	0.63	1.10	0.71	1.72

**Table A4:** Weighted simple logistic regression estimates for covariates from CHMS Cycle 1 with outcome reported psychotropic medicines taken in past month (6 – 19 years of age).

Outcome: Medicine taken							
**Factor (reference category)**	***n***	**Estimate**	**Standard error**	***P*-value**	**Odds ratio (OR)**	**95% CI for odds ratio**	
Child׳s age	2096	0.00	0.03	0.99	1.00	0.94	1.06
BMI – measured	2090	0.05	0.04	0.25	1.05	0.96	1.15
Gender (males)	2096	0.79	0.26	0.01	2.21	1.25	3.93
Education (secondary school or less)	2041	0.67	0.59	0.28	1.95	0.53	7.14
Income (low/lower middle)	1936	0.30	0.57	0.61	1.35	0.39	4.70
Prenatal smoking (yes)	1043	0.87	0.6	0.17	2.40	0.64	9.01
Born any time before due date (yes)	1053	0.76	0.21	0.004	2.14	1.34	3.42
Special neonatal unit care (yes)	1073	−0.07	0.49	0.89	0.93	0.32	2.77
No. of days in neonatal unit	1072	0.00	0.05	0.94	1	0.89	1.11
Hours of sleep per night	2096	−0.2	0.06	0.008	0.82	0.72	0.94
Birth weight	1045	0.00	0.00	0.03	1.00	1.00	1.00
Breast feeding (3 months or longer)	1043	−0.98	0.59	0.13	0.38	0.10	1.38
Mother׳s age at birth	1071	−0.04	0.05	0.46	0.96	0.86	1.07
Anyone smoking at home (yes)	2091	0.67	0.61	0.30	1.95	0.51	7.55
Fasted (yes)	2096	0.95	0.21	0.01	1.87	1.17	2.98

**Table A5:** Weighted simple logistic regression estimates for covariates from CHMS Cycle 1 with outcome SDQ emotional symptoms (ages 6–17 years).

Outcome: SDQ emotional symptoms							
**Factor (reference category)**	***n***	**Estimate**	**Standard error**	***P*-value**	**Odds ratio (OR)**	**95% CI for odds ratio**	
Child׳s age	1717	0.02	0.02	0.48	1.02	0.97	1.07
BMI – measured	1715	0.06	0.03	0.05	1.06	1.00	1.13
Gender (males)	1717	−0.44	0.17	0.03	0.65	0.44	0.95
Education (Secondary school or less)	1676	0.65	0.29	0.04	1.92	1.03	3.61
Income (low/lower middle)	1649	0.34	0.26	0.22	1.40	0.79	2.49
Prenatal smoking (yes)	1045	0.34	0.51	0.53	1.40	0.45	4.34
Born any time before due date (yes)	1054	0.47	0.23	0.07	1.60	0.96	2.67
Special neonatal unit care (yes)	1074	0.47	0.37	0.23	1.60	0.71	3.57
No. of days in neonatal unit	1073	0.02	−0.04	0.98	1.00	0.96	1.04
Hours of sleep per night	1717	−0.2	0.07	0.02	0.82	0.70	0.95
Birth weight	1046	0	0	0.12	1.00	1.00	1.00
Breast feeding (3 months or longer)	1044	−0.29	0.23	0.23	0.75	0.46	1.23
Mother׳s age at birth	1072	0	0.01	0.91	1.00	0.97	1.03
Anyone smoking at home (yes)	1713	0.24	0.26	0.38	1.27	0.71	2.27
Fasted (yes)	1717	0.5	0.18	0.02	1.65	1.10	2.47

**Table A6:** Weighted simple logistic regression estimates for covariates from CHMS Cycle 1 with outcome SDQ hyperactivity/inattention (ages 6–17 years).

Outcome: SDQ hyperactivity/inattention							
**Factor (reference category)**	***n***	**Estimate**	**Standard error**	***P*-value**	**Odds ratio (OR)**	**95% CI for odds ratio**	
Child׳s age	1717	−0.05	0.04	0.15	0.95	0.88	1.02
BMI – measured	1715	0.00	0.02	0.84	1.00	0.96	1.04
Gender (males)	1717	0.51	0.14	0.004	1.66	1.22	2.24
Education (secondary school or less)	1676	0.52	0.26	0.08	1.68	0.94	2.99
Income (low/lower middle)	1649	0.52	0.20	0.02	1.68	1.09	2.58
Prenatal smoking (yes)	1045	0.9	0.28	0.009	2.47	1.32	4.61
Born any time before due date (yes)	1054	−0.07	0.14	0.63	0.93	0.69	1.27
Special neonatal unit care (yes)	1074	−0.1	0.36	0.79	0.90	0.41	2.02
No. of days in neonatal unit	1073	0.02	0.95	0.95	1.00	0.96	1.04
Hours of sleep per night	1717	−0.04	0.08	0.68	0.97	0.81	1.16
Birth weight	1046	0	0	0.88	1.00	1.00	1.00
Breast feeding (3 months or longer)	1044	−0.41	0.18	0.04	0.66	0.45	0.98
Mother׳s age at birth	1072	−0.02	0.02	0.29	0.98	0.94	1.02
Anyone smoking at home (yes)	1713	0.78	0.31	0.03	2.18	1.11	4.31
Fasted (yes)	1717	0.02	0.22	0.92	1.02	0.63	1.67

**Table A7:** Weighted simple logistic regression estimates for covariates from CHMS Cycle 1 with outcome SDQ total difficulties (ages 6–17 years).

Outcome: SDQ total difficulties							
**Factor (reference category)**	***n***	**Estimate**	**Standard error**	***P*-value**	**Odds ratio (OR)**	**95% CI for odds ratio**	
Child׳s age	1717	−0.05	0.05	0.31	0.95	0.86	1.06
BMI – measured	1715	0.04	0.03	0.30	1.04	0.97	1.11
Gender (males)	1717	0.26	0.24	0.29	1.30	0.77	2.19
Education (secondary school or less)	1676	0.42	0.54	0.45	1.52	0.46	5.02
Income (low/lower middle)	1649	0.69	0.30	0.04	1.99	1.02	3.89
Prenatal smoking (yes)	1045	1.15	0.43	0.02	3.17	1.24	8.09
Born any time before due date (yes)	1054	0.32	0.20	0.13	1.38	0.90	2.13
Special neonatal unit care (yes)	1074	0.35	0.24	0.18	1.42	0.83	2.42
No. of days in neonatal unit	1073	−0.00	0.01	0.85	1.00	0.98	1.02
Hours of sleep per night	1717	−0.09	0.12	0.48	0.92	0.70	1.20
Birth weight	1046	0.00	0.00	0.65	1.00	1.00	1.00
Breast feeding (3 months or longer)	1044	−0.45	0.17	0.02	0.64	0.43	0.93
Mother׳s age at birth	1072	−0.03	0.03	0.35	0.97	0.91	1.04
Anyone smoking at home (yes)	1713	0.77	0.33	0.04	2.16	1.04	4.50
Fasted (yes)	1717	0.37	0.28	0.21	1.45	0.78	2.69

**Table B1:** Weighted multiple logistic regression estimates from CHMS Cycle 1 with reported ADD/ADHD (6–19 years of age).

**Exposure**	**Covariate**	**Estimate**	**SE**	***P*-value**		**Odds ratio**	**95% CI for odds ratio**	
BPA	Intercept	−2.96	0.72	0.0018				
	ln(creatinine)	−0.28	0.18	0.1626		0.76	0.51	1.14
	ln(BPA)	0.26	0.18	0.6089		1.29	0.86	1.94
	Smoking at home (yes)	0.84	0.34	0.0312		2.32	1.10	4.90
	Child׳s age	0.05	0.05	0.3131				
	Income (low/low-middle)	0.67	0.31	0.0533		1.95	0.99	3.83
	Gender (males)	−0.17	0.63	0.7911	*Gender*: *males vs*. *females*^a^	*3.06*	*1.67*	*5.61*
	BMI	−0.10	0.03	0.0152		0.91	0.84	0.98
	ln(lead)	0.83	0.26	0.0087		2.28	1.29	4.04
	Gender*child׳s age	0.10	0.05	0.0593	*Age*: *males*	*1.16*	*1.06*	*1.27*
					*Age*: *females*	*1.05*	*0.96*	*1.15*
						
Blood lead	Intercept	−4.13	0.40	<0.0001				
	ln(lead)	0.87	0.27	0.0080		2.39	1.32	4.32
	Smoking at home (yes)	0.93	0.35	0.0215		2.54	1.18	5.46
	Gender (males)	1.08	0.32	0.0064		2.93	1.45	5.94
	Income (low/low-middle)	0.76	0.38	0.0674		2.15	0.94	4.91
						
MBP	Intercept	−2.67	0.68	0.0029				
	ln(creatinine)	0.35	0.25	0.1869		1.42	0.82	2.48
	ln(MBP)	−0.21	0.18	0.2637		0.81	0.55	1.20
	Income (low/lower middle)	−0.24	0.81	0.7697	*Income*: *low/low*-*middle vs*. *middle/upper (males*)	*3.61*	*1.30*	*10.03*
	Gender (males)	0.44	0.41	0.3151	*Income*: *low*/*low-middle vs. middle-upper* (*females*)	*0.78*	*0.10*	*6.15*
	ln(Lead)	1.00	0.27	0.0046		2.71	1.47	5.00
	Income*gender	1.53	0.83	0.0971	*Males vs. females* (*low*/*low*-*middle*)	*7.11*	*1.12*	*44.92*
					*Males vs*. *females* (*middle*/*upper*)	*1.55*	*0.69*	*3.46*
						
MBzP	Intercept	−4.04	0.87	0.0007				
	ln(creatinine)	−0.28	0.24	0.2707		0.76	0.45	1.29
	ln(MBzP)	0.39	0.21	0.0912		1.48	0.93	2.36
	Child׳s age	0.05	0.06	0.4167				
	Income (low/low-middle)	0.79	0.44	0.1013		2.21	0.83	5.84
	Gender (males)	−1.07	0.67	0.1390	*Males vs*. *females*^a^	*2.81*	*1.45*	*5.45*
	ln(lead)	0.84	0.25	0.0066		2.33	1.33	4.06
	BMI	−0.08	0.04	0.0685		0.92	0.84	1.01
	Child׳s age*gender	0.17	0.06	0.0135	*Child׳s age*: *males*	*1.24*	*1.07*	*1.44*
					*Child׳s age*: *females*	*1.05*	*0.94*	*1.18*
						
MCPP	Intercept	−3.81	0.46	<0.0001				
	ln(creatinine)	0.22	0.23	0.3578		1.25	0.75	2.07
	ln(MCPP)	−0.02	0.14	0.8977		0.98	0.72	1.34
	Income (low/low-middle)	0.90	0.53	0.1187		2.45	0.76	7.84
	Gender (males)	1.04	0.38	0.0187		2.83	1.23	6.50
	ln(lead)	0.94	0.27	0.0053		2.56	1.41	4.66
						
MEHHP	Intercept	−2.66	0.84	0.0090				
	ln(creatinine)	0.43	0.28	0.1517		1.53	0.83	2.82
	ln(MEHHP)	−0.34	0.24	0.1771		0.71	0.42	1.20
	Income (low/low-middle)	0.89	0.53	0.1180		2.44	0.77	7.78
	Gender (males)	1.01	0.37	0.0203		2.74	1.21	6.21
	ln(lead)	0.99	0.28	0.0050		2.70	1.45	5.05
						
MEHP	Intercept	−3.63	0.53	<0.0001				
	ln(creatinine)	0.29	0.25	0.2749		1.34	0.77	2.33
	ln(MEHP)	−0.16	0.14	0.2830		0.85	0.62	1.17
	Income (low/low-Middle)	0.92	0.53	0.1070		2.51	0.79	7.99
	Gender (males)	1.03	0.38	0.0210		2.81	1.21	6.56
	ln(lead)	0.97	0.28	0.0048		2.64	1.44	4.85
						
MEOHP	Intercept	−2.82	0.82	0.0054				
	ln(creatinine)	0.42	0.29	0.1808		1.52	0.80	2.89
	ln(MEOHP)	−0.34	0.25	0.1935		0.71	0.41	1.22
	Income (low/low-middle)	0.89	0.53	0.1173		2.44	0.77	7.78
	Gender (males)	0.99	0.37	0.0208		2.70	1.20	6.08
	ln(lead)	0.99	0.28	0.0051		2.70	1.44	5.05
						
MEP	Intercept	−2.70	0.99	0.0193				
	ln(creatinine)	0.48	0.40	0.2587		1.61	0.67	3.88
	ln(MEP)	−0.29	0.23	0.2299		0.75	0.45	1.24
	Income (low/low-middle)	0.93	0.52	0.1031		2.52	0.80	7.95
	Gender (males)	0.97	0.36	0.0218		2.64	1.19	5.90
	ln(lead)	0.96	0.28	0.0059		2.61	1.40	4.86

a Odds ratio calculated at average value of Child׳s age

**Table B2:** Weighted multiple logistic regression estimates from CHMS Cycle 1 with report of a learning disability (6–19 years of age).

**Exposure**	**Covariate**	**Estimate**	**SE**	***P*-value**		**Odds ratio**	**95% CI for odds ratio**	
BPA	Intercept	−3.03	0.28	<0.0001				
	ln(creatinine)	0.10	0.11	0.3623		1.11	0.87	1.41
	ln(BPA)	0.09	0.17	0.6089		1.09	0.76	1.57
	Smoking at home (yes)	0.50	0.19	0.0252		1.65	1.08	2.53
	Gender (males)	0.57	0.26	0.0467		1.78	1.01	3.12
	ln(lead)	1.10	0.36	0.0116				
	Income (low/lower-middle)	0.66	0.35	0.0869	*Income*: *low/low*-*middle vs*. *middle*/*upper*^a^	*2.44*	*1.31*	*4.52*
	ln(lead)*Income	−1.27	0.45	0.0163	*ln*(*lead*): *low/low*-*middle*	*0.85*	*0.38*	*1.86*
					*ln*(*Lead*): *middle*/*upper*	*3.00*	*1.47*	*6.12*
						
Blood lead	Intercept	−3.07	0.26	<0.0001				
	ln(lead)	1.02	0.35	0.0138				
	Smoking at home (yes)	0.57	0.18	0.0088		1.76	1.19	2.61
	Income (low/low-middle)	0.64	0.33	0.0791	*Income*: *low/low-middle vs. middle/upper*^a^	*2.38*	*1.34*	*4.24*
	Gender (males)	0.68	0.29	0.0381		1.98	1.05	3.75
	ln(lead) * income	−1.23	0.45	0.0195	*ln*(*lead*): *low*/*low*-*middle*	*0.81*	*0.37*	*1.81*
					*ln*(*lead*): *middle*/*upper*	*2.78*	*1.40*	*5.51*
						
MBP	Intercept	−1.38	0.72	0.0825				
	ln(creatinine)	0.48	0.14	0.0064		1.61	1.18	2.21
	ln(MBP)	−0.23	0.16	0.1797		0.79	0.56	1.13
	Gender (males)	0.66	0.29	0.0422		1.93	1.03	3.62
	Income (low/lower Middle)	−0.78	0.40	0.0790		0.46	0.19	1.11
	ln(lead)	0.85	0.28	0.0102		2.35	1.28	4.31
						
MBzP	Intercept	−2.96	0.60	0.0005				
	ln(creatinine)	0.15	0.15	0.3396		1.16	0.84	1.61
	ln(MBzP)	0.22	0.16	0.2017		1.24	0.87	1.77
	Gender (males)	0.70	0.30	0.0383		2.02	1.05	3.89
	Income (low/low-Middle)	−0.70	0.41	0.1184		0.50	0.20	1.23
	ln(lead)	0.80	0.28	0.0144		2.23	1.21	4.08
						
MCPP	Intercept	−2.42	0.43	0.0002				
	ln(creatinine)	0.33	0.15	0.0556		1.38	0.99	1.93
	ln(MCPP)	0.17	0.16	0.3235				
	Gender (males)	0.97	0.32	0.0115	*Males vs. females*^b^	*2.08*	*1.15*	*3.76*
	Income (low/low-Middle)	−0.74	0.41	0.0951		0.48	0.19	1.16
	ln(lead)	0.83	0.27	0.0111		2.29	1.26	4.17
	ln(MCPP) * gender	−0.28	0.09	0.0114	*ln*(*MCPP*): *males*	*0.89*	*0.65*	*1.21*
					*ln*(*MCPP*): *females*	*1.18*	*0.86*	*1.62*
						
MEHHP	Intercept	−1.32	0.97	0.1986				
	ln(creatinine)	0.48	0.21	0.0415		1.62	1.02	2.55
	ln(MEHHP)	−0.27	0.25	0.2899		0.76	0.44	1.31
	Gender (males)	0.66	0.28	0.0364		1.94	1.05	3.56
	Income (low/low-Middle)	−0.72	0.39	0.0947		0.49	0.20	1.16
	ln(lead)	0.86	0.27	0.0083		2.37	1.31	4.27
						
MEHP	Intercept	−2.03	0.38	0.0003				
	ln(creatinine)	0.39	0.16	0.0344		1.48	1.03	2.11
	ln(MEHP)	−0.17	0.16	0.3283		0.85	0.59	1.21
	Gender (males)	0.68	0.29	0.0366		1.98	1.05	3.71
	Income (low/low-Middle)	−0.75	0.40	0.0882		0.47	0.19	1.14
	ln(lead)	0.85	0.27	0.0096		2.34	1.29	4.26
						
MEOHP	Intercept	−1.45	0.92	0.1421				
	ln(creatinine)	0.47	0.22	0.0510		1.60	1.00	2.58
	ln(MEOHP)	−0.27	0.26	0.3197		0.76	0.43	1.35
	Gender (males)	0.65	0.28	0.0377		1.92	1.05	3.51
	Income (low/low-Middle)	−0.72	0.40	0.0946		0.49	0.20	1.16
	ln(lead)	0.86	0.27	0.0085		2.37	1.31	4.28
						
MEP	Intercept	−1.29	0.69	0.0874				
	ln(creatinine)	0.53	0.24	0.0467		1.70	1.01	2.88
	ln(MEP)	−0.24	0.17	0.1868		0.79	0.54	1.15
	Gender (males)	0.63	0.28	0.0451		1.88	1.02	3.49
	Income (low/low-middle)	−0.76	0.40	0.0837		0.47	0.19	1.13
	ln(lead)	0.84	0.29	0.0142		2.31	1.23	4.34

a Odds ratio calculated at average value of ln(Lead)

b Odds ratio calculated at average value of ln(MCPP)

**Table B3:** Weighted multiple logistic regression estimates from CHMS Cycle 1 with reported psychotropic medicine taken in past month for behavioral problems (6–19 years of age).

**Exposure**	**Covariate**	**Estimate**	**SE**	***P*-value**		**Odds ratio**	**95% CI for odds ratio**	
BPA	Intercept	−2.53	0.44	0.0001				
	ln(creatinine)	−0.17	0.17	0.3345		0.84	0.58	1.22
	ln(BPA)	−0.03	0.15	0.8625				
	Hours slept	−0.20	0.07	0.0119		0.82	0.70	0.95
	Gender (males)	0.54	0.28	0.0826	*Males vs*. *females*^a^	*2.16*	*1.28*	*3.62*
	Fasted (yes)	0.69	0.25	0.0195		1.98	1.14	3.45
	ln(BPA)*gender	0.65	0.21	0.0093	*ln*(*BPA*): *males*	*1.87*	*1.24*	*2.83*
					*ln*(*BPA*): *females*	*0.97*	*0.72*	*1.31*
						
Blood lead	Intercept	−2.19	0.47	0.0007				
	ln(lead)	1.43	0.40	0.0042				
	Gender (males)	0.73	0.33	0.0485		2.08	1.01	4.29
	Fasted (yes)	0.51	0.30	0.1206	*Fasted* (*yes vs. no*)^b^	*2.25*	*1.19*	*4.26*
	ln(lead)*fasted	−1.62	0.55	0.0135	*ln*(*lead*): *fasted*	*0.83*	*0.34*	*2.02*
					*ln*(*lead*): *no fasting*	*4.20*	*1.92*	*9.17*
							
	Hours slept	−0.22	0.07	0.0095		0.80	0.68	0.94
						
MBP	Intercept	−2.43	0.79	0.0103				
	ln(creatinine)	0.18	0.26	0.5067		1.19	0.68	2.11
	ln(MBP)	−0.02	0.22	0.9369		0.98	0.60	1.60
	Hours Slept	−0.20	0.05	0.0010		0.82	0.74	0.90
	Gender (males)	0.92	0.31	0.0138		2.51	1.26	5.01
	Fasted (yes)	0.71	0.23	0.0108		2.04	1.22	3.40
						
MBzP	Intercept	−1.62	0.63	0.0270				
	ln(creatinine)	0.05	0.23	0.8306		1.05	0.63	1.76
	ln(MBzP)	−0.25	0.19	0.2147				
	Hours slept	−0.23	0.06	0.0018		0.80	0.70	0.90
	Gender (males)	−1.14	0.49	0.0415	*Males vs. females*^c^	*2.45*	*1.31*	*4.58*
	Fasted (yes)	0.77	0.25	0.0110		2.16	1.24	3.77
	ln(MBzP)*gender	0.68	0.15	0.0010	*ln*(*MBzP*): *males*	*1.54*	*1.07*	*2.23*
					*ln*(*MBzP*): *females*	*0.78*	*0.54*	*1.13*
						
MCPP	Intercept	−2.90	0.51	0.0001				
	ln(creatinine)	−0.32	0.29	0.2894				
	ln(MCPP)	0.51	0.19	0.0202		1.66	1.10	2.51
	Hours slept	−0.23	0.05	0.0008		0.79	0.71	0.89
	Gender (males)	0.93	0.33	0.0181	*Males vs*. *Females*^d^	*2.42*	*1.27*	*4.61*
	Fasted (yes)	1.34	0.30	0.0010	*Fasted (yes vs. no)*^e^	*2.45*	*1.50*	*3.99*
	ln(creatinine)*gender	0.59	0.29	0.0640				
	ln(MCPP)*fasted	−0.51	0.15	0.0051	*ln(MCPP)*: *fasted*	*1.00*	*0.69*	*1.44*
					*ln*(*MCPP*): *no fasting*	*1.66*	*1.15*	*2.40*
						
MEHHP	Intercept	−2.80	1.05	0.0221				
	ln(creatinine)	−0.28	0.36	0.4473				
	ln(MEHHP)	0.08	0.30	0.7864		1.09	0.56	2.10
	Hours slept	−0.20	0.05	0.0014		0.81	0.73	0.91
	Gender (males)	0.92	0.32	0.0159	*Males vs*. *females*^d^	*2.39*	*1.28*	*4.46*
	Fasted (yes)	0.73	0.22	0.0067		2.08	1.28	3.37
	ln(creatinine)*gender	0.62	0.29	0.0518				
						
MEHP	Intercept	−2.55	0.66	0.0027				
	ln(creatinine)	−0.22	0.38	0.5757				
	ln(MEHP)	0.01	0.32	0.9795		1.01	0.50	2.05
	Hours slept	−0.20	0.05	0.0012		0.82	0.74	0.91
	Gender (males)	0.91	0.33	0.0184	*Males vs*. *females*^d^	*2.37*	*1.26*	*4.48*
	Fasted (yes)	0.71	0.23	0.0099		2.03	1.23	3.34
	ln(creatinine)*gender	0.61	0.30	0.0671				
						
MEOHP	Intercept	−2.77	0.92	0.0120				
	ln(creatinine)	−0.29	0.36	0.4405				
	ln(MEOHP)	0.09	0.30	0.7702		1.09	0.56	2.13
	Hours slept	−0.21	0.05	0.0014		0.81	0.73	0.91
	Gender (males)	0.92	0.32	0.0147	*Males vs. females*^d^	*2.40*	*1.29*	*4.46*
	Fasted (yes)	0.73	0.22	0.0063		2.08	1.29	3.36
	ln(creatinine)*gender	0.63	0.28	0.0486				
						
MEP	Intercept	−3.36	0.82	0.0018				
	ln(creatinine)	−0.35	0.31	0.2853				
	ln(MEP)	0.16	0.13	0.2471		1.17	0.88	1.54
	Hours slept	−0.18	0.05	0.0045		0.84	0.75	0.93
	Gender (males)	0.96	0.34	0.0168	*Males vs*. *females*^d^	*2.50*	*1.30*	*4.80*
	Fasted (yes)	0.71	0.23	0.0097		2.03	1.23	3.33
	ln(creatinine)*gender	0.61	0.30	0.0664				

a Odds ratio calculated at average value of ln(BPA)

b Odds ratio calculated at average value of ln(Lead)

c Odds ratio calculated at average value of ln(MBzP)

d Odds ratio calculated at average value of ln(creatinine)

e Odds ratio calculated at average value of ln(MCPP)

**Table B4:** Weighted multiple logistic regression estimates from CHMS Cycle 1 with outcome SDQ Emotional Symptoms (ages 6–17 years).

**Exposure**	**Covariate**	**Estimate**	**SE**	***P*-value**	**Odds ratio**	**95% CI for odds ratio**	
BPA	Intercept	1.50	1.12	0.2088			
	ln(creatinine)	0.20	0.13	0.1416	1.23	0.92	1.63
	ln(BPA)	0.07	0.05	0.1682	1.07	0.97	1.19
	Hours slept	−0.27	0.10	0.0183	0.77	0.62	0.95
	Gender (males)	−0.41	0.11	0.0032	0.66	0.52	0.84
	Fasted (yes)	0.49	0.21	0.0424	1.63	1.02	2.61
	Child׳s age	−0.08	0.03	0.0415	0.92	0.86	1.00
					
Blood lead	Intercept	−0.22	0.75	0.7758			
	ln(lead)	0.08	0.21	0.7208	1.08	0.68	1.71
	Gender (males)	−0.40	0.18	0.0508	0.67	0.45	1.00
	Hours slept	−0.19	0.08	0.0370	0.83	0.70	0.99
	Fasted (yes)	0.53	0.25	0.0553	1.70	0.99	2.95
	Education (secondary school or less)	0.57	0.23	0.0324	1.78	1.06	2.97
					
MBP	Intercept	−0.99	0.63	0.1426			
	ln(creatinine)	0.35	0.17	0.0614	1.42	0.98	2.04
	ln(MBP)	−0.14	0.16	0.4087	0.87	0.61	1.24
	Gender (males)	−0.32	0.16	0.0631	0.72	0.51	1.02
					
MBzP	Intercept	1.92	1.06	0.0962			
	ln(creatinine)	0.38	0.17	0.0431	1.47	1.01	2.13
	ln(MBzP)	−0.11	0.08	0.2049	0.90	0.75	1.07
	Gender (males)	−0.29	0.15	0.0792	0.75	0.53	1.04
	Hours slept	−0.24	0.10	0.0386	0.79	0.63	0.99
	Child׳s age	−0.08	0.04	0.0503	0.92	0.84	1.00
					
MCPP	Intercept	1.56	1.11	0.1875			
	ln(creatinine)	0.34	0.18	0.0833	1.41	0.95	2.08
	ln(MCPP)	−0.06	0.08	0.4643	0.94	0.78	1.13
	Gender (males)	−0.29	0.16	0.0890	0.75	0.53	1.05
	Hours slept	−0.23	0.10	0.0447	0.79	0.63	0.99
	Child׳s age	−0.08	0.04	0.0637	0.92	0.85	1.01
					
MEHHP	Intercept	−1.69	0.43	0.0023			
	ln(creatinine)	0.20	0.10	0.0654	1.22	0.98	1.52
	ln(MEHHP)	0.05	0.11	0.6388	1.05	0.83	1.33
	Gender (males)	−0.30	0.15	0.0764	0.74	0.53	1.04
					
MEHP	Intercept	−1.49	0.16	<0.0001			
	ln(creatinine)	0.25	0.10	0.0336	1.28	1.02	1.60
	ln(MEHP)	−0.02	0.09	0.8602	0.98	0.80	1.21
	Gender (males)	−0.30	0.15	0.0755	0.74	0.53	1.04
					
MEOHP	Intercept	−1.68	0.38	0.0011			
	ln(creatinine)	0.20	0.10	0.0750	1.22	0.98	1.53
	ln(MEOHP)	0.06	0.11	0.6145	1.06	0.83	1.34
	Gender (males)	−0.30	0.16	0.0796	0.74	0.53	1.04
					
MEP	Intercept	−1.56	0.27	0.0001			
	ln(creatinine)	0.22	0.12	0.0840	1.25	0.96	1.62
	ln(MEP)	0.01	0.07	0.8631	1.01	0.87	1.18
	Gender (males)	−0.30	0.15	0.0716	0.74	0.53	1.03

**Table B5:** Weighted multiple logistic regression estimates from CHMS Cycle 1 with outcome SDQ Hyperactivity/Inattention (ages 6–17 years).

**Exposure**	**Covariate**	**Estimate**	**SE**	***P*-value**		**Odds ratio**	**95% CI for odds ratio**	
BPA	Intercept	−1.96	0.14	<0.0001				
	ln(creatinine)	−0.08	0.12	0.5184		0.92	0.71	1.20
	ln(BPA)	0.27	0.05	0.0003				
	Smoking at home (yes)	0.91	0.24	0.0031	*Smoking at home* (*yes vs*. *no*)^a^	*1.96*	*1.23*	*3.11*
	ln(lead)	0.94	0.16	0.0001		2.56	1.79	3.65
	ln(BPA)*smoking at home	−0.67	0.21	0.0079	*ln(BPA)*: *smoking at home*	*0.67*	*0.46*	*1.00*
					*ln(BPA)*: *no smoking at home*	*1.31*	*1.18*	*1.46*
						
Blood lead	Intercept	−2.06	0.20	<0.0001				
	ln(lead)	0.85	0.18	0.0005		2.33	1.59	3.43
	Gender (males)	0.36	0.18	0.0705		1.44	0.96	2.14
	Smoking at home (Yes)	0.74	0.36	0.0620		2.10	0.96	4.62
								
MBP	Intercept	−2.40	0.73	0.0070		0.09	0.02	0.45
	ln(creatinine)	0.05	0.20	0.7879		1.06	0.69	1.62
	ln(MBP)	0.16	0.21	0.4626	*Smoking at home* (*yes vs. no*)^b^	*1.59*	*0.94*	*2.71*
	ln(lead)	0.96	0.20	0.0006		2.61	1.67	4.07
	Smoking at home (yes)	2.48	0.92	0.0204				
	ln(MBP)*smoking at home	−0.58	0.28	0.0628	*ln*(*MBP*): *smoking at home*	*0.66*	*0.39*	*1.12*
					*ln*(*MBP*): *no smoking at home*	*1.17*	*0.78*	*1.76*
						
MBzP	Intercept	−2.25	0.30	<0.0001				
	ln(creatinine)	−0.02	0.11	0.8288		0.98	0.77	1.23
	ln(MBzP)	0.16	0.09	0.1047		1.17	0.96	1.43
	ln(lead)	0.97	0.19	0.0003		2.63	1.74	3.99
						
MCPP	Intercept	−1.97	0.22	<0.0001				
	ln(creatinine)	−0.05	0.13	0.7145		0.95	0.71	1.27
	ln(MCPP)	0.20	0.10	0.0821		1.22	0.97	1.53
	ln(lead)	0.95	0.20	0.0006		2.58	1.67	4.00
						
MEHHP	Intercept	−1.89	0.32	0.0001				
	ln(creatinine)	0.07	0.12	0.6030		1.07	0.81	1.40
	ln(MEHHP)	0.04	0.08	0.5976		1.04	0.88	1.23
	ln(lead)	0.98	0.20	0.0004		2.68	1.74	4.12
						
MEHP	Intercept	−1.68	0.17	<0.0001				
	ln(creatinine)	0.12	0.12	0.3375		1.13	0.86	1.49
	ln(MEHP)	−0.05	0.11	0.6440		0.95	0.75	1.20
	ln(lead)	0.99	0.20	0.0004		2.70	1.73	4.20
						
MEOHP	Intercept	−1.79	0.31	0.0001				
	ln(creatinine)	0.08	0.12	0.5150		1.09	0.83	1.43
	ln(MEOHP)	0.02	0.08	0.8472		1.02	0.85	1.21
	ln(lead)	0.99	0.20	0.0004		2.68	1.73	4.15
						
MEP	Intercept	−1.50	0.33	0.0008				
	ln(creatinine)	0.15	0.12	0.2101		1.17	0.90	1.51
	ln(MEP)	−0.07	0.06	0.3207		0.94	0.82	1.08
	ln(lead)	0.99	0.20	0.0005		2.69	1.72	4.20

a Odds ratio calculated at average value of ln(BPA)

b Odds ratio calculated at average value of ln(MBP)

**Table B6:** Weighted multiple logistic regression estimates from CHMS Cycle 1 with outcome SDQ Total Difficulties (ages 6–17 years).

**Exposure**	**Covariate**	**Estimate**	**SE**	***P*-value**	**Odds ratio**	**95% CI for odds ratio**	
BPA	Intercept	−2.36	0.18	<0.0001			
	ln(creatinine)	−0.18	0.15	0.2686	0.84	0.60	1.17
	ln(BPA)	0.13	0.08	0.1118	1.14	0.96	1.36
	Smoking at home (yes)	0.57	0.28	0.0680	1.76	0.95	3.27
	ln(lead)	0.81	0.25	0.0080	2.24	1.29	3.87
					
Blood lead	Intercept	−2.25	0.16	<0.0001			
	ln(lead)	0.77	0.22	0.0049	2.16	1.33	3.51
	Smoking at home (yes)	0.74	0.38	0.0739	2.10	0.92	4.81
					
MBP	Intercept	−2.45	0.95	0.0250			
	ln(creatinine)	−0.04	0.26	0.8961	0.97	0.54	1.72
	ln(MBP)	0.09	0.25	0.7353	1.09	0.62	1.91
	ln(lead)	0.84	0.22	0.0029	2.32	1.43	3.76
					
MBzP	Intercept	−3.15	0.39	<0.0001			
	ln(creatinine)	−0.20	0.19	0.3213	0.82	0.54	1.25
	ln(MBzP)	0.31	0.11	0.0189	1.37	1.06	1.76
	ln(lead)	0.82	0.24	0.0056	2.26	1.34	3.82
					
MCPP	Intercept	−2.19	0.29	<0.0001			
	ln(creatinine)	−0.02	0.25	0.9427	0.98	0.57	1.69
	ln(MCPP)	0.06	0.20	0.7691	1.06	0.68	1.66
	ln(lead)	0.85	0.23	0.0034	2.33	1.41	3.84
					
MEHHP	Intercept	−1.72	0.77	0.0457			
	ln(creatinine)	0.11	0.28	0.7079	1.11	0.60	2.05
	ln(MEHHP)	−0.11	0.18	0.5451	0.89	0.60	1.33
	ln(lead)	0.87	0.25	0.0051	2.38	1.38	4.10
					
MEHP	Intercept	−1.92	0.31	0.0001			
	ln(creatinine)	0.12	0.24	0.6314	1.13	0.66	1.93
	ln(MEHP)	−0.16	0.18	0.3910	0.86	0.58	1.26
	ln(lead)	0.88	0.24	0.0035	2.41	1.43	4.07
					
MEOHP	Intercept	−1.69	0.70	0.0347			
	ln(creatinine)	0.12	0.28	0.6654	1.13	0.61	2.10
	ln(MEOHP)	−0.14	0.19	0.4678	0.87	0.57	1.32
	ln(lead)	0.87	0.25	0.0049	2.39	1.38	4.12
					
MEP	Intercept	−2.25	0.48	0.0007			
	ln(creatinine)	0.00	0.20	0.9975	1.00	0.64	1.55
	ln(MEP)	0.03	0.10	0.7580	1.03	0.83	1.29
	ln(lead)	0.86	0.25	0.0061	2.36	1.35	4.11

**Table C1:** Summary of Multiple Logistic Regression Results for Parent or Self-Reported Outcomes from the Canadian Health Measures Survey Cycle 1 (Weighted) for All Children.

**ADD/ADHD**		**Learning disability**		**Medicine taken**	
**Contaminant**	**Ages 6–19 yrs OR (95% CI)**	**Contaminant**	**Ages 6–19 yrs OR (95% CI)**	**Contaminant**	**Ages 6–19 yrs OR (95% CI)**
**BPA**	1.29 (0.86–1.94)	**BPA**	1.09 (0.76–1.57)	**BPA**	M: 1.87 (1.24–2.83)
					F: 0.97 (0.72–1.31)^a^
**MBP**	0.81 (0.55–1.20)	**MBP**	0.79 (0.56–1.13)	**MBP**	0.98 (0.60-1.60)
					
**MBzP**	1.48 (0.93–2.36)	**MBzP**	1.24 (0.87–1.77)	**MBzP**	M: 1.54 (1.07–2.23)
					F: 0.78 (0.54–1.13)^a^
					
**MCPP**	0.98 (0.72–1.34)	**MCPP**	M: 0.89 (0.65–1.21)	**MCPP**	FS: 1.00 (0.69-1.44)
			F: 1.18 (0.86–1.62)^a^		
					NFS: 1.66 (1.15-2.40)^a^
**MEHHP**	0.71 (0.42.1.20)	**MEHHP**	0.76 (0.44–1.31)	**MEHHP**	1.09 (0.56–2.10)
**MEHP**	0.85 (0.62–1.17)	**MEHP**	0.85 (0.59–1.21)	**MEHP**	1.01 (0.50–2.05)
**MEOHP**	0.71 (0.41–1.22)	**MEOHP**	0.76 (0.43–1.35)	**MEOHP**	1.09 (0.56–2.13)
**MEP**	0.75 (0.45–1.24)	**MEP**	0.79 (0.54–1.15)	**MEP**	1.17 (0.88–1.54)
					
**Blood lead**	2.39 (1.32–4.32)	**Blood lead**	LI: 0.81 (0.37–1.81)	**Blood lead**	FS: 0.83 (0.34–2.02)
			HI: 2.78 (1.40–5.51)^a^		
					NFS: 4.20 (1.92–9.17)^a^

a Interaction terms significant at 5% level between contaminant and other covariates. M: males; F: females; LI: low income; HI; higher income; FS: fasting sample; NFS: non-fasting sample; Models adjusted for covariates as determined by a stepwise multiple regression procedure.

**Table C2:** Summary of multiple regression results for SDQ outcomes from the Canadian health measures survey cycle 1 (weighted) for all children 6–17 years.

**SDQ: Total difficulties**		**SDQ: Emotional symptoms**		**SDQ: Hyperactivity/inattention**	
**Contaminant**	**Ages 6–17 yrs OR (95% CI)**	**Contaminant**	**Ages 6–17 yrs OR (95% CI)**	**Contaminant**	**Ages 6–17 yrs OR (95% CI)**
**BPA**	1.14 (0.96–1.36)	**BPA**	1.07 (0.97–1.19)	**BPA**	ETS: 0.67 (0.46–1.00)
					NETS: 1.31 (1.18-1.46)^a^
**MBP**	1.09 (0.62–1.91)	**MBP**	0.87 (0.61–1.24)	**MBP**	1.59 (0.94–2.71)
**MBzP**	1.37 (1.06–1.76)	**MBzP**	0.90 (0.75–1.07)	**MBzP**	1.17 (0.96–1.43)
**MCPP**	1.06 (0.68–1.66)	**MCPP**	0.94 (0.78–1.13)	**MCPP**	1.22 (0.97–1.53)
**MEHHP**	0.89 (0.60–1.33)	**MEHHP**	1.05 (0.83–1.33)	**MEHHP**	1.04 (0.88–1.23)
**MEHP**	0.86 (0.58–1.26)	**MEHP**	0.98 (0.80–1.21)	**MEHP**	0.95 (0.75–1.20)
**MEOHP**	0.87 (0.57–1.32)	**MEOHP**	1.06 (0.83–1.34)	**MEOHP**	1.02 (0.85–1.21)
**MEP**	1.03 (0.83–1.29)	**MEP**	1.01 (0.87–1.18)	**MEP**	0.94 (0.82–1.08)
**Blood lead**	2.16 (1.33–3.51)	**Blood lead**	1.08 (0.68–1.71)	**Blood lead**	2.33 (1.59–3.43)

a Interaction terms significant at 5% level between contaminant and other covariates. ETS: smoking in home; NETS: no smoking in home; Models adjusted for covariates as determined by a stepwise multiple regression procedure.

## A. Simple logistic regressions – weighted results for children 6–19 years of age, CHMS cycle 1

See Table A1, Table A2, Table A3, Table A4, Table A5, Table A6, Table A7

## B. Multiple logistic regressions – weighted results for all children

See Table B1, Table B2, Table B3, Table B4, Table B5, Table B6

## C. Summary of multiple logistic regression results – weighted for all children

See Table C1, Table C2

## Footnotes

Transparency document Transparency data associated with this article can be found in the online version at doi:10.1016/j.dib.2016.06.017.
