# Supplementary material for: Processed data for CHMS 2007–2009: Bisphenol A, phthalates and lead and learning and behavioral problems in Canadian children 6–19 years of age
Source: Data Brief. 2016 Jun 22;8:784–802. doi: 10.1016/j.dib.2016.06.017 (PMC4956908; doi:10.1016/j.dib.2016.06.017)
Supplement: Supplementary file 1 — Supplementary material [file mmc1.zip › Conflict of Interest Form BOYLAN.pdf]

This statement is signed by all the authors to indicate agreement that the above information is true and correct (*a photocopy of this form may be used if there are more than 10 authors*):

Author's name (typed)	Author's signature	Date
Tye E. Arbuckle		
Karelyn Davis		
Khrista Boylan	[illegible]	May 25/16.
Mandy Fisher		
Jingshan Fu		
