# Supplementary material for: Processed data for CHMS 2007–2009: Bisphenol A, phthalates and lead and learning and behavioral problems in Canadian children 6–19 years of age
Source: Data Brief. 2016 Jun 22;8:784–802. doi: 10.1016/j.dib.2016.06.017 (PMC4956908; doi:10.1016/j.dib.2016.06.017)
Supplement: Supplementary file 1 — Supplementary material [file mmc1.zip › Jason Fu COI.pdf]

Conflicts of Interest Statement DIB-D-16-00298

Manuscript title: Processed Data for CHMS 2007-2009: Bisphenol A, Phthalates and Lead and Learning and Behavioral Problems in Canadian Children 6 – 19 years of age

The authors whose names are listed immediately below certify that they have NO affiliations with or involvement in any organization or entity with any financial interest (such as honoraria; educational grants; participation in speakers' bureaus; membership, employment, consultancies, stock ownership, or other equity interest; and expert testimony or patent-licensing arrangements), or non-financial interest (such as personal or professional relationships, affiliations, knowledge or beliefs) in the subject matter or materials discussed in this manuscript.

Author names:

Tye E. Arbuckle, Karelyn Davis, Khrista Boylan, Mandy Fisher, and Jingshan Fu

The authors whose names are listed immediately below report the following details of affiliation or involvement in an organization or entity with a financial or non-financial interest in the subject matter or materials discussed in this manuscript. Please specify the nature of the conflict on a separate sheet of paper if the space below is inadequate.

Author names:

This statement is signed by all the authors to indicate agreement that the above information is true and correct (*a photocopy of this form may be used if there are more than 10 authors*):

Author's name (typed)	Author's signature	Date
Tye E. Arbuckle		
Karelyn Davis		
Khrista Boylan		
Mandy Fisher		
Jingshan Fu	Jingshan Fu	18/05/2016
